# Investigation of Mutations in Exon 14 of *SH3TC2* Gene and Exon 7 of *NDRG1* Gene in Iranian Charcot-Marie-Tooth Disease Type 4 (CMT4D) Patients

**Published:** 2020

**Authors:** Rahmaneh Sadat MOOSAVI, Niloofar JAHANGIR SOOLTANI, Massoud HOUSHMAND

**Affiliations:** 1 Science and Research Branch of Islamic Azad University, Islamic Republic of Iran, Niloofar Jahangir Soltani; 2 Department of Medical Biotechnology, National Institute of Genetic Engineering and Biotechnology, Tehran, Iran.; 3 Department of National Institute of Genetic Engineering and Biotechnology, Tehran, Iran.

**Keywords:** SH3TC2 gene, NDRG1 gene, CMT4D, Charcot-Marie-tooth disease, Iran

## Abstract

**Objectives:**

Charcot-Marie-tooth disease type 4 (CMT4D) is an autosomal recessive form of Charcot-Marie-tooth disease with an earlier age of onset and greater severity, compared to other types of this disease. CMT4C and CMT4D are the most prevalent subtypes in Mediterranean countries due to the higher rate of consanguineous marriage. In this study, we aimed to identify p.R148X mutation in *NDRG1* gene and p.R1109X mutation in *SH3TC2* gene (responsible for CMT4D and CMT4C, respectively) and to investigate other possible nucleotide changes in exon 14 of *SH3TC2* gene and exon 7 of *NDRG1 *gene in an Iranian population.

**Materials & Methods:**

A total of 24 CMT4D patients, who were referred to Iran Special Medical Center, were clinically and electrophysiologically evaluated in this study. DNA was extracted from the patients’ blood samples. Next, polymerase chain reaction (PCR) assay was carried out, and the products were sequenced and analyzed in FinchTV software.

**Results:**

None of the target mutations were found in this study. Sequencing of *SH3TC2* gene showed SNP rs1025476 (g.57975C>T) in 21 (87.5%) patients, including 7 homozygous and 14 heterozygous individuals.

**Conclusion:**

Despite the high rate of mutations in some populations, it seems that they are very rare in Iranian CMT4D patients. Regarding the association of SNP rs1025476 with CMT4D, further assessments are needed to reach a better understanding of genetic markers and their genetic features and to propose better diagnostic and treatment plans for the Iranian population.

## Introduction

Charcot-Marie-tooth disease (CMTD) is recognized as the most common inherited neuromuscular disorder. It is a gradually progressive disease with an approximate prevalence of 1/2500 people (1). Symptoms of this disease include muscle weakness and atrophy, which may appear from the first to the third decade of life. Genetically, it is a heterogeneous disease with an autosomal dominant, autosomal recessive (AR), or X-linked inheritance (2). 

In recent decades, classification of CMTD has become more complex (3), and its inheritance modes (2, 4, 5) and involved nerves vary in each type. Responsible mutations have been identified in more than 80 genes. Generally, different mutations cause different modes of inheritance (6-8). Charcot-Marie-tooth disease type 4 (CMT4D) is an AR form of demyelinating CMTD. This disease is characterized by an early onset, usually before the age of 2-3 years, and rapid clinical progression, which leads to more distal limb deformities (9, 10).

In recent studies, it has been suggested that mutations in *SH3TC2* gene, which are responsible for CMT4C, are the most common contributing factors for CMT4D not only in the Mediterranean region, but also in European and North American countries (9). *SH3TC4* gene, also known as* KIAA1985* gene, encodes a protein, which is expressed in the peripheral nerves of Schwann cells (11, 12). This protein is required for proper myelination and integrity of the node of Ranvier in the peripheral nervous system (6). According to recent studies, lack of *SH3TC1* gene in recycling endosomes is an underlying molecular defect, leading to CMT4C (13). This gene includes 17 encoding exons and is located on chromosome 5 (5q32). It is clear that a defect in this gene causes myelination defects. 

*NDRG1* gene mutation leads to the development of hereditary motor and sensory neuropathy Lom type, also known as CMT4D (14). CMT4D is characterized by Schwann cell dysfunction, associated with early and severe axonal loss due to axon-glial interaction failure (9, 14). Recent studies have concluded that impaired Schwann cell trafficking fails to meet the considerable demands of nerve growth and may be involved in the pathogenetic mechanism of *NDRG1* deficiency. One of the mutations is located on codon 148 of *NDRG1* gene (15, 16). *NDRG1* gene, which is ubiquitously expressed, contributes to growth arrest, cell differentiation, and possibly signaling protein shuttling between the cytoplasm and the nucleus. This gene is located on chromosome 8q24.3, with a high level of expression in Schwann cells (14).

In the present study, we aimed to investigate CMT4C and CMT4D with an AR mode of inheritance, which show greater severity and earlier age of onset, compared to other types of CMTD (9, 17). Different mutations are responsible for CMT4D. Generally, ten different subclasses have been recognized so far, each responsible for CMT4A–J (9). Since ARCMT is more frequent in countries with a high rate of consanguineous marriage (17), we aimed to analyze two mutations of p.R148X in *NDRG1* gene and p.R1109X in *SH3TC2* gene, which can both create stop codons, responsible for CMT4D and CMT4C, respectively. Considering the higher rate of consanguineous marriage in some regions of Iran, we may assume that these mutations play a significant role in this population. 

Early onset of CMTD subtypes and their severe phenotypes, which are associated with severe polyneuropathy and specific deformations, encouraged us to collect more information about these diseases in the Iranian population. In this study, we aimed to investigate the presence of p.R148X mutation in *NDRG1* gene and p.R1109X mutation in *SH3TC2* gene and to identify other possible mutations and polymorphisms in 24 Iranian CMT4D patients, who were referred to Iran Special Medical Center. (No 4, Ostadnejatolahi St. Tehran, Iran.)

## Materials &Methods 

In this survey, a total of 24 patients (14 males and 10 females; mean age=8.7 years), who were referred to a medical center, were studied under the supervision of neurologists and clinical geneticists for accurate diagnosis of CMT4D. Most patients presented with early-onset demyelinating neuropathy, distal muscular hypotrophy, scoliosis, and other basic features of the disease. No mutation in previously screened *GDAP1* gene, which is another responsible gene for CMT4D (9), was found in the patients. Both parents of all probands were healthy, and informed consent was obtained from the parents or guardians of the patients. 

A blood sample (2 cc) was collected from each patient and preserved in a falcon tube, containing EDTA at -20°C until DNA extraction. Genomic DNA was extracted using Genpajoohan DNA Extraction Kit (Iran) and stored at -20°C. Target DNA fragments of *NDRG1* exon 7 and *SH3TC2* exon 14 were amplified by polymerase chain reaction (PCR) assay with forward and reverse primers (primer sequences and PCR conditions are available upon request). Appropriate synthesis of PCR products was evaluated using 1.5% Agarose gel electrophoresis. 

The search for mutations (p.R148X mutation in *NDRG1* gene and p.R1109X mutation in *SH3TC2* gene) was conducted via Sanger sequencing of PCR products, using the ABI3700 tool (Kosar Kavosh Fanavaron Co., Iran). FinchTV software was also used for analyzing the sequences in order to identify mutations. The sequences were blasted in NCBI website (http://www.ncbi.nlm.nih.gov/blast) and compared with normal sequences. The length of PCR products for analyzing exon 14 of *SH3TC2* gene was 331 bp. To ensure the authenticity of the products, they were compared with a 100-bp DNA ladder via 1.5% Agarose gel electrophoresis. In addition, the length of PCR product was 170 bp for exon 7 of *NDRG1* gene, which was compared with the 50-bp DNA ladder.

## Results

**Figure 1:**
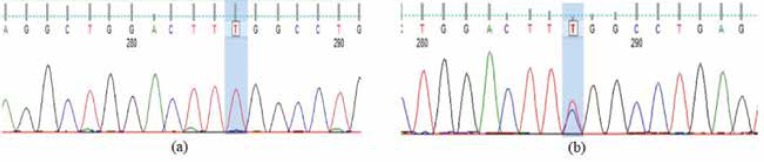
(a) Homozygous g.57975C>T variant in exon 14 of SH3TC2 gene and (b) heterozygous g.57975C>T variant of SH3TC2 gene

Sequence analysis indicated that p.R148X mutation of *NDRG1* gene and p.R1109X mutation of *SH3TC2* gene (both responsible for CMT4D) were not present in the target regions and that the sequences were normal. Based on the sequence analysis of *SH3TC2* gene (exon 14), 21 out of 24 probands (87.5%) showed g.57975C>T variant (c.3327+70C>T), including 14 (58%) heterozygous and 7 (29%) homozygous individuals for this nucleotide mutation (Figure 1). The three remaining patients showed normal sequences. 

Analysis of *NDRG1* sequences (exon 7) indicated that all 24 probands had normal sequences in the evaluated region and that no nucleotide changes were observed. Some sequence analyses were performed for assessment by reverse primers. To ensure the results of sequencing, four samples were randomly considered for assessment by reverse primers (two samples for *SH3TC2* gene and two samples for *NDRG1* gene). The results showed the authenticity of previous results obtained from analyses using forward primers.

## Discussion

According to previous studies, AR-CMTD can be found in people of all races. As multiple studies have indicated, the prevalence of mutations varies in different populations. It is noticeable that in Western countries, the prevalence of AR-CMTD is significantly lower than the dominant form of CMTD (12). Evidence suggests that the prevalence of AR-CMTD is about 10% in Europe and 30-50% in Mediterranean countries (18).

According to a previous study on recessive CMTD, conducted in 2014 in Germany, involvement of genes, such as *GDAP*, *HINT1*, *SH3TC2*, and *NDRG1,* was estimated at 10.9%, 10.3%, 7.5%, and 6.3%, respectively (19). In a previous study on a gypsy population, involvement of these genes, including p.R148X mutation in *NDRG1* gene, was reported in nearly 4.46% of AR-CMTD cases (19). On the other hand, in a study on another gypsy population in Bulgaria, the high frequency of p.R148x mutation was reported, and mutation carriers accounted for 10-16% of the population (20). 

The presence of p.R1109X mutation in *SH3TC2* gene has not been examined separately, and different results have been reported regarding the overall contribution of this mutation. CMT4D (mutation in *NDRG1* gene), a common form of AR-CMTD in gypsy populations, has been reported in European countries. It is the most frequent peripheral neuropathy among Serbian gypsies (21, 22), whereas in Spain, the prevalence rate is different. In addition, CMT4C (mutation in *SH3TC2 *gene) is the most common form of CMT4D in the gypsy population of Spain, followed by CMT4G and CMT4D (23). 

In another study from Spain on 29 gypsy individuals with recessive CMT4D, the prevalence of *SH3TC2* gene mutation (CMT4C) was 57.14%, the prevalence of *HK1* gene mutation (CMT4G) was 25%, and the prevalence of CMT4D was 17.86% (21). Furthermore, a study from South of Italy on 197 CMTD patients revealed that the prevalence of *SH3TC2* gene was only 2% in the general population in the same period (24). Moreover, a study performed in 2016 in Germany showed that the overall prevalence of *SH3TC2* gene was 2.7% in patients with demyelinating CMTD (25).

Although the rate of these mutations is very low in most countries, a high rate has been reported in gypsy populations, as mentioned earlier, which may be attributed to their specific features and genetic differences (20, 26, 27). According to previous studies, the gypsy population was influenced by the bottleneck effect due to the migration of their ancestors from India to Europe. Also, research on genetic markers show that gypsies migrated from India to countries, such as Pakistan, Iran, Turkey, South Armenia, and Europe (28); therefore, genetic merging may have occurred in some traits and genes in these countries. 

Evidence suggests that pathogenic mutations responsible for AR myasthenic syndromes were inherited from the common ancestry history of gypsy, Indian, and Pakistani populations (29). 

Therefore, the incidence of these mutations in the Iranian population is highly expected considering the prevalence of consanguineous marriage in some parts of Iran. Based on studies from Turkey and Spain, one of the target mutations in our study (p.R1109X) had a very distant ancestral origin; this finding shows that mutations occurred in new generations. Moreover, estimation of allelic age indicates that p.R1109X mutation in *SH3TC2* gene was related to the bottleneck effect and probably occurred 225 years ago (between the late 18^th^ century and early 19^th^ century) (21). According to previous studies, these mutations mostly occurred in isolated populations with a high rate of consanguineous marriage. 

Despite the presence of these mutations in other populations from some Mediterranean and European countries, such as Spain and Czechoslovakia, no mutations were found in our study; accordingly, the studied mutations are very rare in non-specific populations. Differences between the findings of our study and studies on other populations can be attributed to differences in the structure, ethnic background, and origin of Iranian population. Considering the limitations in the availability of CMT4 probands, lack of the studied mutations in our population may indicate that other mutations in *SH3TC2 *and* NDRG1 *genes or other causative genes are involved in CMT4C and CMT4D due to the heterogenic features of the disease.

Analysis of the intronic region near exon 14 of *SH3TC2 *gene indicated rs1025476 SNP (g.57975C>T). It should be noted that this SNP was found in a study on a Turkish control population (30). Moreover, since this SNP was found in the majority of proband genes, this mutation may be one of the genetic characteristics of the studied population; such findings can be useful for a better understanding of the genomic features of Iranian population. To clarify the association between this SNP and CMTD, further assessments using case-control studies with a higher number of patients and greater funding are necessary to overcome the limitations of previous studies. 

In Conclusion, By applying more comprehensive methods, such as next generation sequencing, in future studies, we can have a more comprehensive view about this issue. Overall, our findings contribute to the available genetic data regarding CMT4D in the Iranian population. Further genetic analysis can provide a better diagnostic approach for this disease through identification of rare or common genetic features in each specific population.
